# Testing the Insecticidal Activity of Nanostructured Alumina on *Sitophilus oryzae* (L.) (Coleoptera: Curculionidae) Under Laboratory Conditions Using Galvanized Steel Containers

**DOI:** 10.3390/insects9030087

**Published:** 2018-07-23

**Authors:** Guillermo Pablo López-García, Micaela Buteler, Teodoro Stadler

**Affiliations:** 1 Laboratorio de Entomología, Instituto Argentino de Investigaciones de Zonas Áridas (IADIZA), CONICET Mendoza, Mendoza 5500, Argentina; 2 Instituto de Investigaciones en Biodiversidad y Medioambiente (INIBIOMA), CONICET-Universidad Nacional del Comahue, Bariloche 8400, Argentina; butelermica@gmail.com; 3 Laboratorio de Toxicología Ambiental, Instituto de Medicina y Biología Experimental de Cuyo (IMBECU), CONICET Mendoza, Mendoza 5500, Argentina; lpe@mendoza-conicet.gob.ar

**Keywords:** stored grain pests, insecticidal efficacy, nanoparticulate dust, grain damage, frass, progeny

## Abstract

Most stored-grain pest insects increase their population within a relatively short time, causing serious damage to stored products. *Sitophilus oryzae* (L.) is one of the world’s major stored-grain pest insects and was chosen as the model insect for our studies. This study compared the efficacy of three different dusts under laboratory conditions: aluminum dust (nanostructured alumina), DiatomiD^®^, and Protect-It^®^ (commercial diatomaceous earth). Parental survival, grain damage, and progeny production were measured at 250 and 500 ppm in treated wheat. The tests were conducted in 400 mL galvanized steel jars, an experimental model used for the first time to measure the effectiveness of nanostructured alumina, since most studies have been typically performed in small petri dishes. Parental survival obtained was highest in the untreated controls, followed in decreasing order by DiatomiD^®^, Protect-It^®^, and nanostructured alumina (NSA). NSA caused the greatest mortality. All treatments significantly reduced grain weight loss and frass production in wheat infested by *S. oryzae*. The degree of progeny (F1) suppression was directly related to the product and treatment rate, progeny being significantly suppressed by NSA in wheat followed by Protect-It^®^ and DiatomiD^®^. Therefore, NSA had a greater impact on insect population dynamics.

## 1. Introduction

Stored grain insect pests cause damage to grain during storage through their feeding habits and metabolism, producing heat and moisture accumulation, and creating damage hotspots in grain, which result in substantially reduced grain quality by spoilage, reduced germinability, and increased microbial infestation. The extent of grain damage depends on the voraciousness of each insect pest species and the rate of population growth [1,2,3,4]. Most stored-grain pest insects are able to increase their population within a relatively short time, causing serious damage to stored products. Among the stored grain insect pests, *Sitophilus oryzae* (L.) is one of the world’s major stored grain pest insects [5,6].

The growing demand for low impact agriculture has encouraged researchers to search for alternatives outside the framework of organic synthesis, exploring different natural substances such as plant extracts, oils, mineral powders, and ashes, as well as synthetic ultrafine nanoparticle dusts [7,8,9,10,11,12,13,14,15,16,17,18,19,20].

Despite the rapid incorporation of nanotechnology into agriculture and crop protection in the last ten years and its presentation as an innovative tool for pest control, nanotechnology applied to pest management remains a challenge [8,21,22,23,24,25,26,27,28,29]. The discovery of nanostructured alumina (NSA) as an insecticidal dust created new opportunities in pest control with inorganic nanoparticle dusts (nanoinsecticides), due to the low concentration (500 ppm) needed to obtain a mortality rate of 95% in *S. oryzae* after nine days of exposure [14]. However, further studies are needed to determine the potential of NSA as a tool to manage stored grain pests. Among mineral powders or inert dusts (IDs), microparticle powders based on diatomaceous earth (DE) continue to be the most widely studied for control in stored grain, with commercial options available [3,4,30,31,32,33].

Most of the bioassays assessing the efficacy of IDs were conducted in laboratory conditions with DE assessing progeny production (F1) and progeny survival, in addition to grain damage and frass production [1,2,3,4,9,34,35,36,37,38,39]. Furthermore, bioassays with nanoinsecticidal dusts (NDs), like nanostructured alumina (NSA) and nanosilica, have evaluated lethal concentration (LC) values and insect mortality in laboratory conditions, as well as percentage of mortality at different relative humidity (RH) levels [14,40,41].

With regard to effects on progeny, some authors [9,11,42] assessed the efficacy of DE on progeny of *S. oryzae* under laboratory conditions and measured population growth of *Sitophilus zeamais* Motsch. Arthur [9] found that progeny production was optimal at 27 °C and 75% RH in untreated wheat and lower in Protect-It^®^ treatments. For progeny production in wheat treated with NSA, using standard Petri dishes, Stadler et al. [41] concluded that NSA dust was more effective than Protect-It^®^ in eliminating F1 of *S. oryzae.*

Consistent with the information described above, the initial parental population, percentage of mortality, progeny production in *S. oryzae*, and grain damage (weight loss and frass production) are the main criteria for long term evaluation of the efficacy of insecticidal powders, such as IDs including NDs. So far, efficacy tests using NSA have been based on exposure bioassays performed with small amounts (30 g) of wheat in standard 90 mm Petri dishes and a standard number of initial adult insects [14,41]. The objective of this study was to assess the insecticidal efficacy of NSA and DE (DiatomiD^®^ and Protect-It^®^) under laboratory conditions increasing the size of the experimental unit by using galvanized steel jars to allow for greater amounts of wheat and greater numbers of insects. Galvanized steel is the same material used in the manufacture of silos [43,44,45].

## 2. Materials and Methods

### 2.1. Insect Culture

All *S. oryzae* individuals used in the bioassays were obtained from a colony raised in the Laboratory of Environmental Toxicology, IMBECU CONICET CCT-Mendoza in 250 mL flasks on wheat kernels var. Baguette 501 (NIDERA). The insects were reared in incubators at 27 ± 1 °C, 70 ± 5% relative humidity (RH) and continuous darkness. RH was maintained by using a container with saturated solution of sodium chloride in water [46]. Temperature and humidity inside the chambers were monitored with HOBO data recorders (Onset Computer, Bourne, MA, U.S.).

### 2.2. Insecticides Dust Used for Bioassays

Nanostructured alumina (Al_2_O_3_) was obtained via the glycine-nitrate combustion synthesis technique using a redox mixture with glycine as the fuel and aluminum nitrate nonahydrate as the oxidizer [47]. The apparent density of the powder was calculated at 0.108 g/cm^3^. Particle size analysis showed a bi-modal size distribution and platelet morphology, with a 45-nm plate thickness. According to electromagnetic properties, NSA particles tend to form agglomerates that appear as larger particles depending on their shape, size, and surface reactivity [48]. The largest particle diameter measured was 1.5 µm with a nanosized distribution of smaller particle diameters at 350 nm [26,41].

Two commercial formulations of DE, DiatomiD^®^ and Protect-It^®^, were used in the bioassays. Protect-It^®^ is a mixture of 87% amorphous silicon dioxide originated from freshwater DE with 10% silica gel and other compounds such as 3% Al_2_O_3_, 1% Fe_2_O_3_, and less than 1% CaO, MgO, TiO_2_, P_2_O_3_. Median particle size was reported to be 5.4 mm; apparent density was 0.20 g/cm^3^ [4,30]. This inert dust was obtained from Hedley Technologies Ltd. (Grand Junction, CO, U.S.). DiatomiD^®^ contains over 85% amorphous SiO^2^, 4% Al_2_O_3_, 1% Fe_2_O_3_, 1% CaO, and 2–4% water. It is a natural diatomaceous earth product obtained from fossilized sedimentary deposits of microalgae (diatoms) from San Juan, Argentina, with a density of 0.54 g/cm^3^ and without any substances added.

### 2.3. Laboratory Efficacy Tests: Adult Survival, Grain Damage and Progeny Production Assessment

Efficacy of NSA was assessed in terms of exposure toxicity bioassay, grain consumption, and progeny production determined by the multiplication rate (MR) at two different insecticide concentrations, and this nanoinsecticide was contrasted with two commercial insecticidal powders: DiatomiD^®^ and Protect-It^®^.

### 2.4. Experimental Design and Bioassay Conditions

Tests were conducted in 400-mL galvanized steel cylindrical container (7 cm in diameter and 12 cm tall with a sealed base). The structural material used is similar to that used in silos found in the field [43,44]. The wheat used as the bioassay substrate was acclimatized in an incubator at 27 ± 1 °C and 70 ± 5% RH for one week. The moisture content of the wheat was 11 ± 0.5% as determined by a moisture analyzer (model MX-50; A & D Company, Ltd. (Tokyo, Japan)). Concentrations of 250 ppm and 500 ppm of NSA, Protect-It^®^, and DiatomiD^®^ were prepared in wheat kernels and homogenized by shaking for 3 min to evenly distribute the dusts throughout the entire grain mass. A total of 200 g of wheat from each treatment were placed in the steel jars and each was replicated five times. Five galvanized steel jars containing 200 g of untreated wheat were used as control. Two hundred 1–2 week-old unsexed adult *S. oryzae* were placed in each galvanized steel jars. After introduction of the test insects, the top of each container was sealed with a layer of 0.5 mm mesh screen. The treatments were maintained in the incubator at 27 ± 1 °C and 70 ± 5% RH, in complete darkness. Adult survival was assessed on days 7, 14, and 21 after continuous exposure to treated wheat. On day 21, after reaching 100% mortality with NSA at 250 ppm, all insects were removed and jars were returned to the incubator for an additional 6 weeks (42 days) in order to determine the progeny production effects of NSA, DiatomiD^®^, and Protect-It^®^ (Table 1). Each F1 was then removed from the different treatments, counted, and tabulated. Multiplication rate was calculated as: MR = (Number of individuals in F1/Number of parental individuals) × 1/*n* − 1, where (*n*) = 3 weeks, the time during which the parental generation was subjected to treated and untreated wheat. After the 21-day incubation, the wheat from each jar was collected and sieved through a 0.6 mm mesh to sift out the frass produced by parental population of insects. The sifted frass and remaining kernel fraction were weighed separately. Damage assessment was performed by measuring the weight of the sieved powder and that of the grains without powder (final weight). Percent weight loss in treated and control sets was calculated on a fresh weight basis using equation: (Initial weight − final weight/initial weight) × 100.

### 2.5. Data and Statistical Analyses

Data from adult mortality, grain damage, and progeny production were processed using statistical software SAS 9.3 (SAS Institute Inc., Cary, NC, USA) [49]. Mortality, grain damage, and progeny data were tested by analysis of variance (ANOVA) using a mixed-model design (PROC MIXED) with mortality, grain damage, and progeny as the response variables and concentration and treatment as the main effects. In the mortality assessment, exposure time was the repeated measure variable. LS means were compared using the Tukey procedure.

## 3. Results

### 3.1. Parental Survival (Adults)

**Figure 1:**
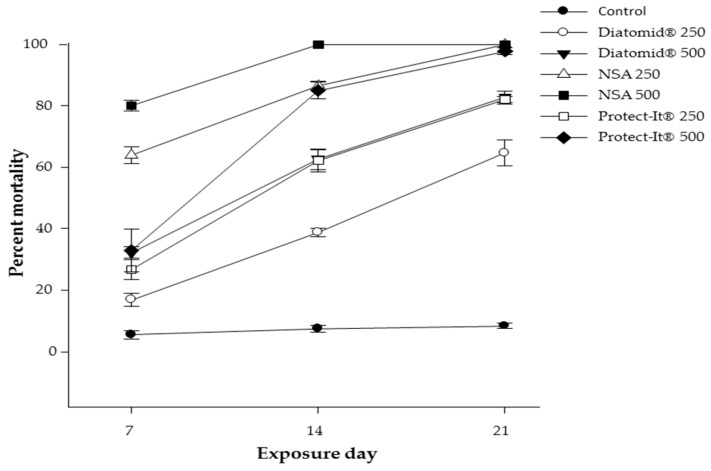
Average percent mortality of *Sitophilus oryzae* (L.) in wheat treated with 250 and 500 ppm of nanostructured alumina (NSA), DiatomiD^®^, and Protect-It^®^ at 27 ± 1 °C and 75% relative humidity (RH) at different exposure times.

There was a significant treatment effect on overall adult mortality over time (F = 135.72; df = 2.78; *p* < 0.0001), as well as exposure time effect (F = 191.64; df = 2.78; *p* < 0.0001). Mortality increased with product concentration (F = 56.89; df = 2. 78; *p* < 0.0001) (Table 1, Figure 1). Parental survival was greatest in the untreated control, followed by DiatomiD^®^, Protect-It^®^, then NSA. NSA caused the greatest mortality, followed by Protect-it^®^ and DiatomiD^®^ (Figure 1). Parental survival after a seven-day exposure to 250 ppm treated wheat ranged between 83% in DiatomiD^®^, 73.2% in Protect-it^®^, and 36% in NSA. After 14 days exposure, overall mean survival in wheat treated with 250 ppm was 61% in DiatomiD^®^, 37% in Protect-it^®^, and 13% in NSA. After 21 days of exposure, NSA dust at 250 ppm achieved 100% mortality, whereas mortalities for DiatomiD^®^ and Protect-it^®^ were 65% and 82%, respectively (Figure 1). Parental survival of *S. oryzae* in 500 ppm treated wheat on day seven ranged between 68% for DiatomiD^®^, 67% for Protect-it^®^, and 20% for NSA. After 14 days of exposure to wheat treated with 500 ppm of dust, parental survival ranged between 17% for DiatomiD^®^, 2.2% for Protect-it^®^, and 0% for NSA.

### 3.2. Grain Damage (Grain Weight Loss and Frass Production)

Treatments with NSA, Protect-It^®^, and DiatomiD^®^ significantly reduced grain weight loss and frass in *S. oryzae* infested wheat (Table 2). A significant treatment effect was observed on wheat consumed by adults on day 21 (F = 194.5; df = 6.28; *p* < 0.0001) as well as on frass produced (F = 74.93; df = 6.28; *p* < 0.0001). The lowest frass production was attained in wheat treated with NSA and Protect-it^®^ at 500 ppm, followed by NSA and Protect-it^®^ at 250 ppm. The amount of frass was significantly greater in DiatomiD^®^ treatments, followed by the control treatment. As to wheat consumption, NSA and Protect-it^®^ treatments did not differ from one another, showing a significant reduction compared to the control and DiatomiD^®^ treatments.

### 3.3. F1 Production (Transgenerational Effects of NSA on Sitophilus Oryzae)

The mean number of F1 weevils emerging from the untreated control wheat grains after a 21-day infestation period was 2171, approximately 10 times higher than the parental population, and significantly greater than emergence from the other treatments (Table 2). The degree of progeny suppression was directly related to the product and treatment rate, with progeny in wheat being significantly suppressed by NSA (F = 456.4; df = 6.28; *p* < 0.0001). All three treatments generally caused a significant dose-dependent reduction in progeny production relative to the control (Table 2). The greatest progeny reduction was achieved after 21 days of contact of *S. oryzae* with wheat treated with NSA at 500 ppm. Progeny production in wheat treated with 500 ppm of Protect-it^®^ and DiamotiD^®^ was significantly lower, revealing a much lower impact on *S. oryzae* population dynamics.

## 4. Discussion

The results of the present study were consistent with those obtained by Stadler and colleagues [14,41], where the insecticidal activity of NSA was greater than that of DE (DiatomiD^®^ and Protect-It^®^) (Table 1 and Table 2, Figure 1). The low mortality observed in treated wheat with DiatomiD^®^ at concentrations of 250 and 500 ppm was expected considering that recommended doses are greater than 1000 ppm [3,50]. Different studies suggest that the efficacy of IDs depends on grain moisture and relative ambient humidity (RH) [1,2,26,41,51]. Even though this experiment used a greater density of insects and the material of the containers simulated real steel grain containers, the mortality attained was similar to that obtained in previous studies testing inert dusts in 90-mm Petri dishes [14,41]. We found that a similar amount of time was required for the dusts to attain 100% mortality in steel jars (cylindrical container) at a concentration of 500 ppm compared to previous studies using Petri dishes at 27 ± 1 °C and 75% RH [41]. This indicates good performance of NSA even under high insect pressure as well as at high ambient humidity conditions.

Wheat consumption and frass production by *S. oryzae* were significantly reduced by NSA as well as by Protect-it^®^ compared to the control and DiatomiD^®^ treatments. Regarding the effect on F1 of the products tested, NSA reduced progeny production (F1) of *S. oryzae* to a greater extent than Protect-It^®^ and DiatomiD^®^ at the same concentrations and exposure time. This was expected as NSA caused greater adult mortality and the multiplication rate values (F1) obtained were inversely proportional to mortality in the parental cohort. Total percentage of parental survival of *S. oryzae* was significantly different for the control, NSA, Protect-it^®^, and DiatomiD^®^ in the different treatments after 7, 14, and 21 days of exposure. Survival was greatest in the untreated control and decreased significantly with exposure to DiatomiD^®^, followed by Protect-It^®^ and then NSA, which had the lowest survival (Table 1 and Table 2, Figure 1). Coinciding with Arthur [9], progeny was higher in the untreated control wheat, recording a substantial population growth of more than 10 times the initial parental population, under controlled conditions of 27 °C and 75% RH, after six weeks of incubation (42 days). Although NSA led to mortality of infesting *S. oryzae* at an earlier time than the other products, it was not totally effective at 500 ppm. This is in connection with the 7- to 14-day time to lay eggs before reaching 100% mortality of the parental generation. Multiplication rate values (F1) (Table 2) are inversely proportional to mortality in the parental cohort exposed to the different products and doses tested. In galvanized steel jars, NSA significantly suppressed progeny in wheat, coinciding with results obtained in standard 90 mm Petri dishes [41]. NSA was more effective than DE in reducing the number of insects emerged in F1 at the two concentrations tested. Notably, NSA, like Protect-It^®^ and DiatomiD^®^, generally caused a significant reduction in progeny production, which was dose-dependent in all cases (Table 2). However, NSA had a greater impact on *S. oryzae* population dynamics. In connection with the increased mortality of weevils in NSA exposure bioassays, seed water content as well as RH impact the survival of individuals [9,26,41]. Therefore, practical application of NSA to protect stored products is limited by environmental conditions and the treatment of grain should be performed before or shortly after warehousing.

However, due to the low dose of NSA used (125–250 ppm) [14,41], compared with diatomaceous earth (2500–5000 ppm) [1,2,3,4], the dirty grain effect was not visible. Also, no reduced grain flowability was observed in wheat treated with NSA at such small doses (data not shown).

Until now, NSA dust has achieved promising results, although it is not registered for use in stored wheat. We are currently developing toxicity and ecotoxicity tests (OECD) in order to complete the information required for the registration process. As this is a new nanoparticle dust tested in laboratory conditions, at the moment we do not have cost values for its industrial production. Further studies assessing the efficacy on NSA in the field are warranted.

## 5. Conclusions

In this study, insecticidal activity of NSA on *S. oryzae* was measured using galvanized steel containers, under laboratory conditions. Parental survival, grain damage, and progeny produced by insects were dose-dependent. The results obtained did not differ significantly from those obtained with standard 90 mm Petri dishes at 27 ± 1 °C and 70 ± 5% RH [41], suggesting that standard bioassays are appropriate to determine efficacy of inert dusts. The present study demonstrated, once again, that NSA is highly effective in the control of *S. oryzae* infesting stored wheat, showing greater efficacy than the diatomaceous earth-based products Protect-it^®^ and DiatomiD^®^. NSA had a greater impact on population dynamics as it caused greater adult mortality as well as a greater reduction in progeny production. Based on these results, NSA is a good candidate as a tool to manage stored grain insect pests and should be studied further.

## Figures and Tables

**Table 1:** Overall mortality of *Sitophilus oryzae* from laboratory efficacy tests-dry dust application of nanostructured alumina (NSA) and diatomaceous earth (DE).

Treatment (ppm)	Percent Mortality	Letters
Control (0)	8.4	A
DiatomiD^®^ (250)	64.6	B
Protect-It^®^ (250)	82	C
DiatomiD^®^ (500)	82.7	C
Protect-It^®^ (500)	97.8	D
NSA (250)	100	D
NSA (500)	100	D

Bioassay endpoint = 21 days, *n* = 200 adults per replicate, five replicates conducted, substrate = 200 g of untreated and treated wheat kernels. Means in the same column followed by the same letter are not significantly different at *p* < 0.05.

**Table 2:** Mean wheat weight loss, frass production, and number of adult of *Sitophilus oryzae* in F1 after 21 days of parental generation exposure and 42 days of development at 27 ± 1 °C and 75% relative humidity (RH). MR is the obtained multiplication rate.

Treatment (ppm)	% Wheat Weight Loss	Frass Grams (SE)	N° Adults F1 (SE)	Multiplication Rate of F1
Control	17.46	1.85 (0.15)	2171 (49.1)	0.30
DiatomiD^®^ 250	1.64	0.92 (0.02)	1363 (15.6)	0.24
DiatomiD^®^ 500	0.25	0.46 (0.12)	816 (47.2)	0.17
Protect-It^®^250	0.19	0.32 (0.04)	574 (41.3)	0.12
Protect-It^®^500	0.23	0.27 (0.05)	434 (26.4)	0.09
NSA 250	0.15	0.22 (0.03)	306 (14.6)	0.05
NSA 500	0.12	0.13 (0.002)	214 (14.6)	0.01

Ppm (Parts per million); SE (Standard error).
